# Efficacy and Safety of Compound Kushen Injection on Patients with Advanced Colon Cancer: A Meta-Analysis of Randomized Controlled Trials

**DOI:** 10.1155/2017/7102514

**Published:** 2017-11-12

**Authors:** Lixiu Yu, Ying Zhou, Yu Yang, Furong Lu, Yeqin Fan

**Affiliations:** ^1^Department of Pharmacy, Union Hospital, Tongji Medical College, Huazhong University of Science and Technology, Wuhan 430022, China; ^2^Department of Integrated Traditional Chinese and Western Medicine, Union Hospital, Tongji Medical College, Huazhong University of Science and Technology, Wuhan 430022, China; ^3^School of Pharmacy, Tongji Medical College, Huazhong University of Science and Technology, Wuhan 430030, China

## Abstract

**Objective:**

The efficacy and safety of Compound Kushen Injection (CKI) on advanced colon cancer remain controversial. We undertook a systematic meta-analysis of randomized controlled clinical studies on this issue.

**Methods:**

A comprehensive literature search was conducted by searching the following electronic databases: PubMed, Cochrane, Chinese Biological Medical disc, Chinese National Knowledge Infrastructure, and Wan-Fang Database in China by the end of January 31, 2017, without language restriction. Meta-analysis was performed by using the random effects model to estimate the summary odd ratio (OR) with 95% confidence interval (CI) according to the study design. Stata 12.0 software was used for data analysis. The heterogeneity, sensitivity, and publication bias were assessed, respectively.

**Results:**

A total of 14 trials met the inclusion criteria in present meta-analysis. The results suggested that CKI combined with chemotherapeutic drugs was favorable for the treatment of advanced colon cancer and could improve the patients' life quality. Funnel plot analysis and Egger's test suggested that there was not significant publication bias, and the sensitivity analysis indicated stable results.

**Conclusion:**

The current evidence suggested that CKI is favorable to improve the efficacy of chemotherapeutic drugs in patients with advanced colon cancer.

## 1. Introduction

Colon cancer is a malignant tumor of digestive tract and is considered to be the third most common cancer in the world [1, 2]. It is generally known that balanced diet is important to maintain health. While, in recent years, with the improvement of people's living standard, most people eat more greasy food than coarse grains or dietary fiber in daily life, which increased the risk of colon cancer [3, 4]. In addition, the symptom of early colon cancer is relatively hidden and not easy to diagnose that many patients are being diagnosed at advanced stage [5, 6]. FOLFOX (oxaliplatin, 5-FU, and leucovorin) adjuvant chemotherapy remains the backbone of colon cancer patients. It can improve survival rate of colon cancer patients [7, 8]. However, chemotherapeutic drugs often decreased the immune function of the patients and produced a variety of adverse drug reactions (ADRs) such as nausea, vomiting, thrombocytopenia, and liver function damage, which seriously affected the patient's life of quality [9, 10]. The efficacy and safety of chemotherapeutic drugs need to be improved.

In recent years, an increasing number of studies have proved that the traditional Chinese medicine (TCM), especially in combination with chemotherapeutic drugs, played an important role in improving the effectiveness and immunomodulation [11, 12]. Furthermore, TCM can reduce the toxic and side effects caused by chemotherapy medicament [13–15]. Compound Kushen Injection (CKI) is one of the commonly used TCMs in China, which is mainly extracted from two different Chinese herbs: Kushen* (Radix sophorae flavescentis)* and Baituling* (Rhizoma smilacis glabrae)* [16, 17]. As CKI contains various active components such as matrine, oxymatrine, and sophoridine, it exhibits a variety of pharmacological activities, including anti-inflammatory, antiallergic, antiviral, antifibrotic, and especially anticancer activities [18, 19]. In addition, previous studies confirmed that CKI had the effect of heat-clearing, dampness-expelling, blood cooling, detoxification, and sanjie analgesic effect [20]. It is extensively used for the treatment of pain and bleeding with malignant tumor such as liver cancer [21], lung cancer [22], and gastrointestinal cancer [18]. The suggested mechanisms of action are increasing protein expression, inhibiting vascular endothelial growth factor expression in neoplastic tissues, killing cell activity, inducing tumor cells to normal cell differentiation, and promoting cell apoptosis and so on [23–26]. Meanwhile, published studies have suggested that CKI had synergetic antitumor effects in different ways and target different molecular pathways. It can improve metabolism and blood circulation, enhance the patient's immunity, relieve pains, and reduce some disoperation of chemotherapeutics [27, 28].

As we know, side effects such as leukopenia, gastrointestinal adverse reactions, alopecia, and bone marrow depressions are common after chemotherapy [16]. How to reduce the adverse reactions and improve the quality of life of patients has become a concern of researchers. Investigators have proved the attenuation effect of the CKI to the toxic and side effects during chemotherapy [29]. And it has been proved that the effective components of matrine and oxymatrine are small molecule drug used in humans for the treatment of tumors with little adverse effects [30]. In addition, CKI did not produce damaging effects on normal cells and could increase white blood cell count and enhance immune function compared to other chemotherapeutic drugs [31, 32]. A systematic meta-analysis was conducted to evaluate the effect of CKI on relieving cancer-related pain [16]. The findings showed that CKI plus chemotherapy could relieve cancer-related pain better than chemotherapy except for colorectal cancer. However, for all we know, there was not a meta-analysis to evaluate efficacy and safety of CKI for advanced colon cancer. In this study, we aimed to carry out a meta-analysis to assess the efficacy and safety of CKI on this issue.

## 2. Methods

### 2.1. Literature Search

We searched the online databases including PubMed, Cochrane, Chinese Biological Medical disc (CBM), Chinese National Knowledge Infrastructure (CNKI), and Wan-Fang Database in China without language restrictions until January 31, 2017. The key words used in this search were as follows: “colon cancer/colonic carcinoma (MeSH Terms)”, “Kushen”,* “Radix sophorae flavescentis”*, “Baituling”,* “Rhizoma smilacis glabrae”*, “CKI” and “chemotherapy”. Furthermore, hand searching techniques were also used to identify the appropriate studies and just full length original articles were included. Studies which have only reported abstracts were excluded.

### 2.2. Inclusion and Exclusion Criteria

The inclusion criteria were as follows: patients with advanced colon cancer (stage III-IV colon cancer) which was confirmed by pathologic or histologic investigation and who had no hypertension, hyperthyroidism, malignant tumor, or other diseases; patients received CKI combined with chemotherapeutic drugs versus patients just received chemotherapeutic drugs; randomized controlled trials (RCTs); outcome assessment included effect analysis such as complete remission, partial remission, quality of life (assessed by Karnofsky Performance Status (KPS) scoring criteria), and adverse drug reactions. The exclusion criteria were (i) reviews, nonclinical studies; (ii) non-RCTs; (iii) controlled interventions with other Chinese herbs therapies or acupuncture; and (4) no outcome measurements or duplicated citations.

### 2.3. Data Extraction

Data was extracted independently by two investigators (Yeqin Fan and Lixiu Yu) according to the above inclusion and exclusion criteria. Any discrepancy between the reviewers was solved by discussion. General information of the eligible studies including first author's name, published year, cases of participants in CKI combined with chemotherapeutic drugs group and chemotherapeutic drugs alone group, age, chemotherapy regimen, immune function, and effects of CKI treatment were selected from each article. Specific data such as effect analysis and ADR were also extracted carefully.

### 2.4. Data Synthesis and Analysis

Statistical analyses were conducted by using Stata 12.0 software (StataCorp LP, College Station, TX). The efficacy and safety of CKI on patients with advanced colon cancer were measured by pooled odd ratio (OR) with 95% confidence interval (CI) at the end of treatment course. The Cochran* Q *statistic and *I*^2^ were introduced to evaluate the heterogeneity of eligible studies. If substantial heterogeneity exists (*I*^2^ > 50%), the estimated outcomes across eligible studies were calculated by random effect model [44]. Otherwise fixed effect model was preferred [45]. And a statistical test with *p* < 0.05 was considered significant. For detection of potential publication bias, funnel plots and Egger's tests were introduced (*p* < 0.05 suggested statistical significance) [46]. Meanwhile, to assess the robustness of the combined effects, sensitivity analysis was also performed, which were performed by sequential removal of each study.

## 3. Results

### 3.1. Search Results

**Figure 1:**
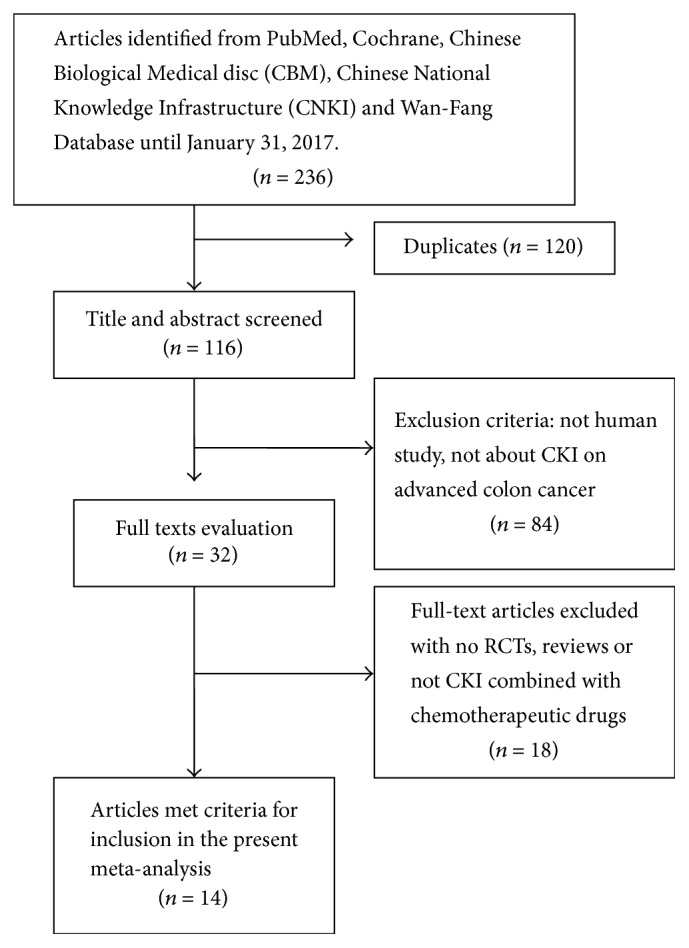
The process of selection of the eligible studies.

The process of selection of the included studies was illustrated in Figure 1. A total of 236 citations were found through online databases and manual searching. After removing the duplications, 116 articles were left. Then 84 studies were excluded by reviewing the titles and abstracts based on predetermined criteria. After further review, 32 articles were chosen for full text evaluation. By thoroughly reading the full text 18 articles were also excluded based on the inclusion criteria. Finally, fourteen published RCTs [27, 29, 31, 33–43] with 1145 advanced colon cancer patients were included in the present meta-analysis.

### 3.2. Characteristics of Identified Studies

The baseline characteristics of all included studies are presented in Table 1. All these clinical trials were RCTs and performed in China. The dates of publication were between 2013 and 2016. The stages of the patients with colon cancer were advanced stages. A total of 11 studies used CKI plus Oxaliplatin (OXA) + Calcium Folinate (CF) + 5-Fluorouracil (5-FU) regimen. The rest of the three studies were used CKI plus OXA + CF, OXA + Raltitrexed (RA), Irinotecan (IR) + CF + 5-FU, respectively. Three studies [31, 36, 39] reported numbers of patients achieved improvement of quality of life. Eleven clinical trials [27, 29, 33–35, 37, 38, 40–43] reported ADR of the patients. The methodological quality evaluation of eligible trials was listed in Table 2.

### 3.3. Meta-Analysis Results

#### 3.3.1. Efficacy of CKI Combined with Chemotherapeutic Drugs

**Figure 2:**
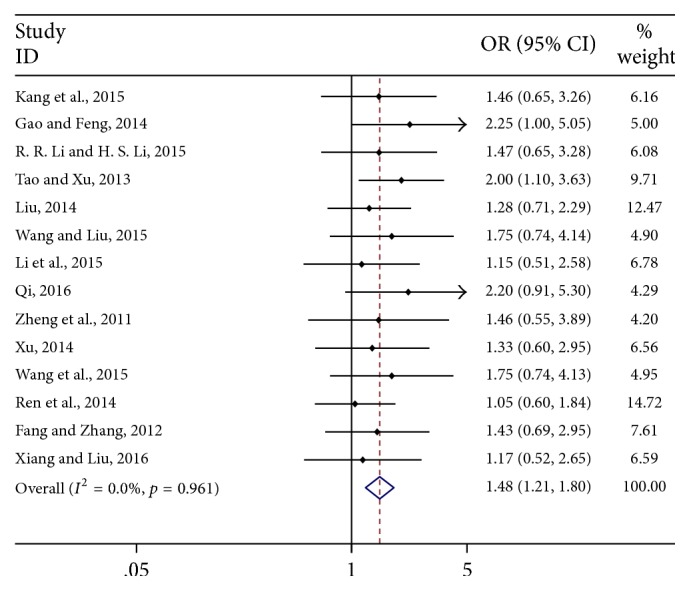
Pooled results of efficacy of CKI combined with chemotherapeutic drugs for advanced colon cancer patients.

The meta-analysis of all the included studies indicated that in the CKI combined with chemotherapeutic drugs group 382 of 581 patients reached complete remission (CR) or partial remission (PR), while 255 of 564 subjects achieved CR or PR in the chemotherapy alone group. Compared with chemotherapy alone, CKI combined with chemotherapy had a favorable effect with OR = 1.48 (95% CI: 1.21–1.80). *I*^2^ = 0%, *p* = 0.961 demonstrated that the heterogeneity across the included studies was not significant and then the fixed effect model was used in this pooled analysis (Figure 2). Furthermore, in the subgroup analysis, we found that CKI combined with chemotherapeutic drugs had significant association with CR and PR. The integrated OR for CR was 1.76 (95% CI: 1.28–2.43, *I*^2^ = 0%, *p* = 0.998), and the pooled OR for PR was 1.35 (95% CI: 1.08–1.70; *I*^2^ = 0%, *p* = 0.885). These findings indicated that CKI in combination with chemotherapeutic drugs had better effects than chemotherapeutic drugs alone.

#### 3.3.2. Life Quality Improvement

**Figure 3:**
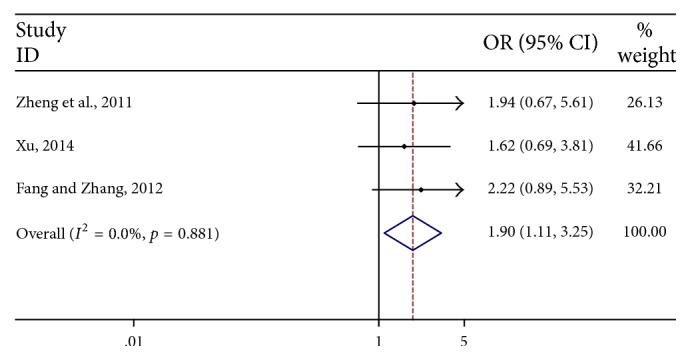
Pooled results of life quality improvement of CKI combined with chemotherapeutic drugs for advanced colon cancer patients.

Three articles [31, 36, 39] reported the quality of life improvement at the end of the treatment. The results indicated that significant heterogeneity (*I*^2^ < 30%) was not found among these articles; thus fixed effect model was applied in this meta-analysis. As shown in Figure 3, the results indicated that CKI combined with chemotherapeutic drugs had an advantage of improving life quality compared with control group (OR = 1.90, 95% CI: 1.11–3.25).

#### 3.3.3. Safety Evaluation

**Figure 4:**
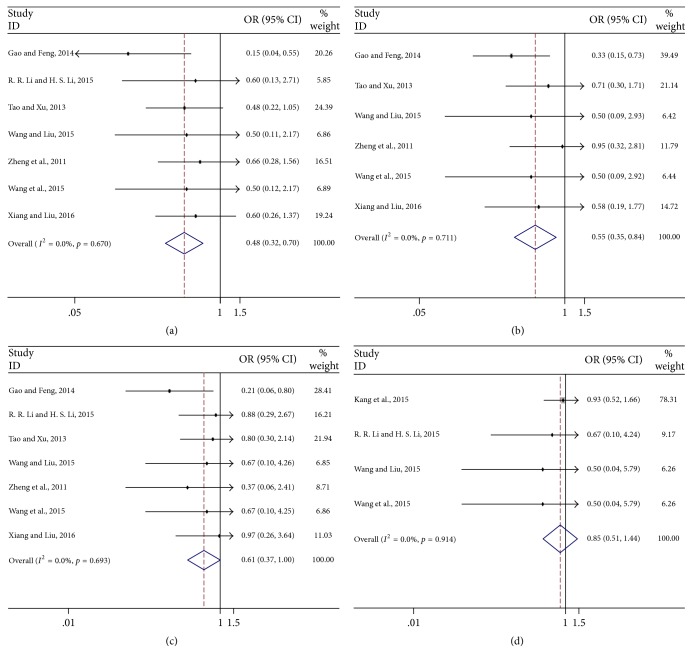
*Meta-analysis CKI combined with chemotherapeutic drugs on the safety of advanced colon cancer patients* ((a) nausea and vomiting; (b) leucopenia; (c) thrombocytopenia; (d) liver damage).

Subgroup meta-analysis of safety evaluation showed that CKI combined with chemotherapeutic drugs could reduce the adverse events for nausea and vomiting, the pooled OR = 0.48 (95% CI: 0.32–0.70) (Figure 4(a)); for leukopenia, OR = 0.55 (95% CI: 0.35–0.84) (Figure 4(b)); for thrombocytopenia, OR = 0.61 (95% CI: 0.37–1.00) (Figure 4(c)), while there was no significant difference for liver damage (OR = 0.85, 95% CI: 0.51–1.44) (Figure 4(d)). In addition, the heterogeneity between the study estimates was not significant (nausea and vomiting*: I*^2^ = 0.0%, *p* = 0.670; leukopenia: *I*^2^ = 0.0%, *p* = 0.711; thrombocytopenia: *I*^2^ = 0.0%, *p* = 0.693; liver damage: *I*^2^ = 0.0%, *p* = 0.914) as shown in Figure 4. For the safety of patients with advanced colon cancer, the results suggested that CKI combined with chemotherapeutic drugs is superior to chemotherapeutic drugs alone.

### 3.4. Publication Bias and Sensitivity Analysis

**Figure 5:**
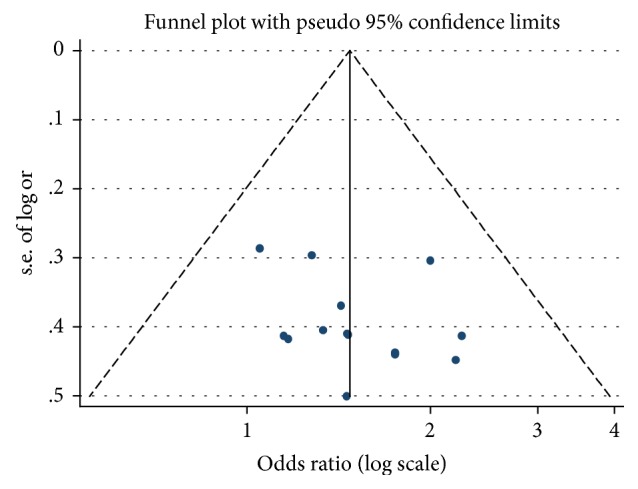
The funnel plot for assessing publication bias.

**Figure 6:**
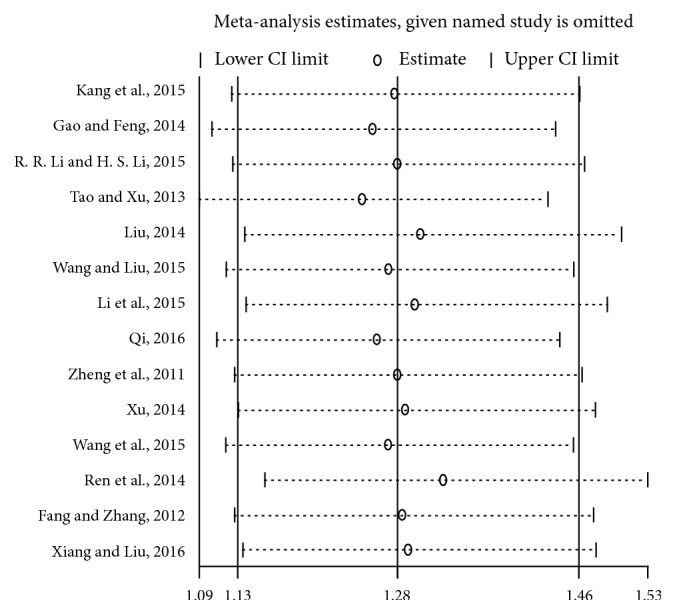
*Sensitivity analysis of the overall ORs*. The results were calculated by omitting each eligible study.

Funnel plot and Egger's test were conducted to evaluate the publication bias of articles. The funnel plots and Egger's test (*p* = 0.267) did not reveal significant evidence of publication bias (Figure 5). Furthermore, to evaluate the influence of each article, sensitivity analysis was performed. From the results we can see a series of pooled ORs with 95% CIs produced similarly before and after eliminating each study at a time, which proved that our results were conservative and robust in Figure 6.

## 4. Discussion

To the best of our knowledge, this study is the first meta-analysis with focus on the efficacy and safety of CKI plus chemotherapeutic drugs in advanced colon cancer patients. A total of 1145 patients were included for the analysis. The pooled results exhibited that CKI combined with chemotherapeutic drugs could be a preferable treatment option for advanced colon cancer patients, because efficacy (OR = 1.48, 95% CI: 1.21–1.80) benefits had been significantly improved. Though the 14 eligible articles are mainly from China, the favorable effect of CKI on advanced colon cancer should not be ignored and underestimated.

Colon cancer had an upward trend of the incidence worldwide [47]. Although surgical treatment has made great progress, but the five-year overall survival of colon cancer is still hovering around 50% [48]. In recent years, chemotherapy can prolong the lives of patients and improve the patients' quality of life [49]; nevertheless, the commonly chemotherapeutic regimens often seriously impair the life quality of the patients and cause serious constraints on the use of chemotherapeutic drugs. It is urgent to seek more optimized combination chemotherapy. CKI plus chemotherapeutic drugs has been widely used for patients with tumors in China for decades. Our subgroup meta-analysis suggested that CKI plus chemotherapeutic drugs could significantly reduce adverse reaction such as nausea and vomiting (OR = 0.48, 95% CI: 0.32–0.70), leucopenia (OR = 0.55, 95% CI: 0.35–0.84), and thrombocytopenia (OR = 0.61, 95% CI: 0.37–1.00) except for liver function damage (OR = 0.85, 95% CI: 0.51–1.44). Furthermore, the quality of life was also significantly improved (OR = 1.90, 95% CI: 1.11–3.25) in CKI combined with chemotherapeutic drugs group. It further strengthened the evidence that CKI could reduce the adverse events of chemotherapy drugs. However, the evidence is limited, and further trials are required to confirm our findings.

As we know, the development and prognosis of malignant tumor are closely related to immune function. T lymphocytes play an important role in the immune system. Sensitized T lymphocytes have specific killing effect on tumor cells. CD4+ T helper cells coordinate B cell differentiation to produce antibody. CD8+ cells could inhibit the synthesis, secretion of antibodies, and proliferation of T cells. The level or proportion of T cell subsets change will cause some changes of immune function. Published studies suggested that active ingredient matrine in CKI can improve the immune function and prolong the survival of patients [18, 50, 51]. Kang et al. demonstrated that the immune function and life quality were significantly improved in patient with advanced colon cancer by CKI plus chemotherapeutic drugs treatment [33, 38]. CD3+, CD4+, and CD4+/CD8+ T lymphocytes percentage was not significantly changed in CKI plus chemotherapeutic drugs, while CD3+, CD4+, and CD4+/CD8+ T lymphocytes percentage significantly decreased after chemotherapeutic drugs alone treatment. The concentrations of IL-2 and TNF-*α* also play an important role in the immune function, which may enhance the ability of tumor controlling for cancer patients [37, 41]. Li et al. proved that CKI plus chemotherapeutic drugs not only can significantly improve the clinical efficacy of patients with advanced colon cancer but also can regulate the immune system function such as increasing the serum level of IL-2, TNF-*α*, and TNF-*γ* [31]. However, as eligible trials were limited, we did not evaluate the effect of CKI combined with chemotherapeutic drugs on immune function of patients with advanced colon cancer in this meta-analysis.

There are several limitations to this study, which need to be concerned. First, although we retrieved current online databases including PubMed, Cochrane, CNKI, CBM, and Wan-Fang Database in China without language restrictions, all the included articles relating to CKI combined with chemotherapeutic drugs versus chemotherapeutic drugs alone for advanced colon cancer patients were only found in China, which indicated that publication bias might exist across studies. Secondly, we found that CKI combined with chemotherapeutic drugs had beneficial effects in the improvement of efficacy and safety in patients with advanced colon cancer. However, the quality of the included RCTs was not high and had relatively small cases, which might cause some bias. Future research should focus on methodologically strong RCTs to determine the potential efficacy of CKI. Thirdly, it was not an individual patient data analysis, and meta-analyses based on published data tended to overestimate treatment effects compared with individual patient data analysis [52]. In addition, the included study lacked a more comprehensive analysis such as adjusting for baseline factors and other differences that existed between the studies from which the data was pooled. Fourthly, though in this study we did not found significant heterogeneity, the chemotherapy regimen and duration of CKI treatment were inconsistent between studies, which increased the risk of heterogeneity. In addition, most of the included studies were performed at hospital among patients with major organ function normal; thus the results may not entirely apply to the general patient population in the community or patients with organ damaged or dysfunction.

On the other hand, there are some highlights in our study. Our meta-analysis systematically and comprehensively examined the efficacy and safety of CKI combined with chemotherapeutic drugs on patients with advanced colon cancer. The favorable treatment of CKI in combination with chemotherapeutic drugs may be highly meaningful for patients with advanced colon cancer. Furthermore, due to comprehensive analysis of all eligible studies, our meta-analysis increases the power and plausibility of the conclusion when compared with previous individual studies.

## 5. Conclusion

In summary, although the quality of the eligible studies is not sufficient, our findings are helpful for clinical practice and treatment for advanced colon cancer. CKI combined with chemotherapeutic drugs is favorable to improve the efficacy and quality of life and increase the safety by reducing the incidence of nausea, vomiting, thrombocytopenia, and leucopenia in patients with advanced colon cancer. However, high-quality studies should be conducted to confirm our findings.

## Figures and Tables

**Table 1:** Baseline characteristic of eligible articles.

Studies	Cases		Effect (CR + PR)		Chemotherapeutic drugs		Age (year)		ADR	Immune function
	T	C	T (CR/PR)	C (CR/PR)	T	C	T	C		
Wang and Liu, 2015 [27]	32	32	21 (3/18)	12 (1/11)	OXA + CF + 5-FU + CKI (12 ml/d)	OXA + CF + 5-FU	21–72	23–71	1, 3, 5, 6, 7	—
Tao and Xu, 2013 [29]	74	74	46 (14/32)	23 (7/16)	OXA + CF + 5-FU + CKI (15 ml/d)	OXA + CF + 5-FU	49–74	49–74	1, 3, 5, 8, 10, 11, 12	—
Li et al., 2015 [31]	26	26	23 (16/7)	20 (13/7)	OXA + CF + 5-FU + CKI (15 ml/d)	OXA + CF + 5-FU	45–68	45–70	—	IL-2, TNF-*α*, INF-*γ*
Kang et al., 2015 [33]	52	52	19 (1/18)	13 (0/13)	OXA + CF + 5-FU + CKI (20 ml/d)	OXA + CF + 5-FU	66.31 ± 7.29	66.31 ± 7.29	2, 4, 6	CD3+, CD4+, CD4+/CD8+, NK
Gao and Feng, 2014 [34]	40	40	27 (8/19)	12 (3/9)	OXA + CF + 5-FU + CKI (15 ml/d)	OXA + CF + 5-FU	31–73	30–75	1, 3, 4, 5, 8, 9	—
R. R. Li and H. S. Li, 2015 [35]	35	35	22 (2/20)	15 (1/14)	IV + CF + 5-FU + CKI (15 ml/d)	IR + CF + 5-FU	41–63	42–64	1, 4, 5, 6, 7	—
Liu, 2014 [36]	52	52	46 (21/25)	36 (17/19)	OXA + CF + 5-FU + CKI (20 ml/d)	OXA + CF + 5-FU	36–78	38–76	—	—
Qi, 2016 [37]	36	36	22 (7/15)	10 (3/7)	OXA + CF + 5-FU + CKI (15 ml/d)	OXA + CF + 5-FU	42–68	43–60	1, 3	—
Zheng et al., 2011 [38]	36	20	21 (3/18)	8 (1/7)	OXA + CF + 5-FU + CKI (15 ml/d)	OXA + CF + 5-FU	40–69	40–69	1, 3, 4, 5, 11	CD3+, CD4+, CD8+, CD4+/CD8+
Xu, 2014 [39]	30	30	24 (12/12)	18 (8/10)	OXA + CF + 5-FU + CKI (15 ml/d)	OXA + CF + 5-FU	45–78	43–78	—	—
Wang et al., 2015 [40]	33	33	21 (3/18)	12 (1/11)	OXA + CF + 5-FU + CKI (15 ml/d)	OXA + CF + 5-FU	52.3 ± 6.2	51.8 ± 5.9	1, 3, 5, 6, 7	—
Ren et al., 2014 [41]	60	60	42 (14/28)	40 (8/32)	OXA + CF + CKI (12 ml/d)	OXA + CF	37–73	33–76	2, 4, 6, 7,	—
Fang and Zhang, 2012 [42]	36	36	30 (20/10)	21 (9/12)	OXA + CF + 5-FU + CKI (12 ml/d)	OXA + CF + 5-FU	17–70	18–72	1, 8	—
Xiang and Liu, 2016 [43]	39	38	18 (7/11)	15 (3/12)	OXA + R + CKI (20 ml/d)	OXA + RA	29–75	31–73	1, 3, 5, 6, 11, 12	—

*Note*. T: Compound Kushen Injection combined with chemotherapeutic drugs group; C: chemotherapeutic drugs alone group; CR: complete remission; PR: partial remission; 1: nausea and vomiting; 2: myelosuppression; 3: leukopenia; 4: digestive tract reaction (Diarrhea); 5: thrombocytopenia; 6: liver injury; 7: renal injury; 8: neurotoxicity; 9: anaemia.; 10: oral mucositis; 11: hypochromia; 12: peripheral phlebitis.

**Table 2:** Methodological quality evaluation of eligible trials.

Studies	Random	Blinding	Incomplete data	Allocation	Selective reporting	Other bias
Wang and Liu, 2015	Yes	No	No	NA	No	NR
Tao and Xu, 2013	Yes	No	No	NA	No	NR
Li et al., 2015	Yes	No	No	NA	No	NR
Kang et al., 2015	Yes	No	No	NA	No	NR
Gao and Feng, 2014	Yes	No	No	NA	No	NR
R. R. Li and H. S. Li, 2015	Yes	No	No	NA	No	NR
Liu, 2014	Yes	No	No	NA	No	NR
Qi, 2016	Yes	No	No	NA	No	NR
Zheng et al., 2011	Yes	No	No	NA	No	NR
Xu, 2014	Yes	No	No	NA	No	NR
Wang et al., 2015	Yes	No	No	NA	No	NR
Ren et al., 2014	Yes	No	No	NA	No	NR
Fang and Zhang, 2012	Yes	No	No	NA	No	NR
Xiang and Liu, 2016	Yes	No	No	NA	No	NR

*Note*. NA: not available; NR: not reported.
